# Neuroendocrine tumor of the breast showing invasive micropapillary features and multiple lymph node metastases

**DOI:** 10.1002/cnr2.1775

**Published:** 2022-12-26

**Authors:** Tomonori Kawasaki, Tomoaki Tashima, Chisako Muramatsu, Akihiro Fujimoto, Yoko Usami, Hitomi Kodama, Jiro Ichikawa, Hirokazu Nagai, Kiyomi Taniyama

**Affiliations:** ^1^ Comprehensive Cancer Center Saitama Medical University International Medical Center Hidaka Japan; ^2^ Breast Oncology Team Saitama Sekishinkai Hospital Sayama Japan; ^3^ Graduate School of Medicine University of Yamanashi Chuo Japan; ^4^ Clinical Research Center National Hospital Organization (NHO) Nagoya Medical Center Aichi Japan; ^5^ Faculty of Data Science Shiga University Hikone Japan; ^6^ Department of Diagnostic Pathology NHO Kure Medical Center and Chugoku Cancer Center Hiroshima Japan

**Keywords:** breast, invasive micropapillary carcinoma, neuroendocrine neoplasm, neuroendocrine tumor

## Abstract

**Background:**

Herein, for the first time, we present a case with mixed invasive micropapillary and neuroendocrine mammary neoplasm.

**Case:**

The patient, a 65‐year‐old postmenopausal woman, had become aware of a tumor in her right breast 11 months prior to presentation at our hospital. The cut surface of the mastectomy specimen contained a well‐circumscribed, multinodular, red‐brown tumor, measuring 15x15x15 cm. Histopathologically, this solid cystic lesion consisted of medullary growth of cancer cells accompanied by a well‐developed vascular network as well as conspicuous hemorrhage. Cancer cell nests of various sizes displayed an “inside‐out” structure surrounded by empty spaces. Most cancer cells were polygonal, though a few were short fusiform‐shaped, and possessed finely granular, eosinophilic cytoplasm and ovoid, fine‐granular nuclei. Eighteen mitotic figures were observed in 10 high‐power fields. Macrometastases, up to 13x8 mm in size, with the same morphological features as the original tumor site, were identified in 3 of 15 dissected right axillary nodes. Immunohistochemically, primary and metastatic cancer cells were diffusely positive for chromogranin A and the estrogen receptor (Allred's total score: 8) and focally reactive for synaptophysin and the progesterone receptor (total score: 5). HER2 and cytokeratin 5/6 were negative, and the MIB‐1 labelling index was 36.2%. MUC1 and EMA lined the stroma‐facing surfaces of the cell membranes, indicating reversed polarity.

**Conclusion:**

Our current patient, who had an invasive breast carcinoma with concomitant neuroendocrine and micropapillary features, developed multiple nodal metastases in association with a large‐diameter tumor showing a luminal B‐like immuno‐profile. Accordingly, meticulous clinical follow‐up remains essential for this uncommon case.

## 1 INTRODUCTION

In the WHO classification of mammary tumors, neuroendocrine neoplasms (NENs) represent less than 1% of invasive breast carcinomas (IBCs) and have recently been classified into two subtypes, that is, neuroendocrine tumor (NET) and neuroendocrine carcinoma, as a cross‐organ disease concept.^1^ Invasive micropapillary carcinoma is a rare special type, accounting for 0.9–2% of all IBCs, showing a characteristic inside‐out growth pattern surrounded by artifactitious clear spaces.^2^ Herein, we present the first documented case, to the best of our knowledge, with an invasive micropapillary mammary NET.

## 2 CASE PRESENTATION

A 65‐year‐old postmenopausal Japanese woman had become aware of a tumor in her right breast 11 months prior to presentation at our hospital. Previous diseases included acute lymphocytic leukemia (9 years earlier), unexplained peritoneal abscess (7 years earlier), and multiple cerebral infarctions (1 year earlier), but her familial history was unclear. Ultrasonography and computed tomography (CT) revealed a huge, cystic right breast tumor with enlarged regional nodes. No other suspected lesions were identified by either systemic CT or bone scintigraphy. The patient underwent fine‐needle aspiration of the mammary lesion, and the cytological diagnosis was carcinoma.

**FIGURE 1:**
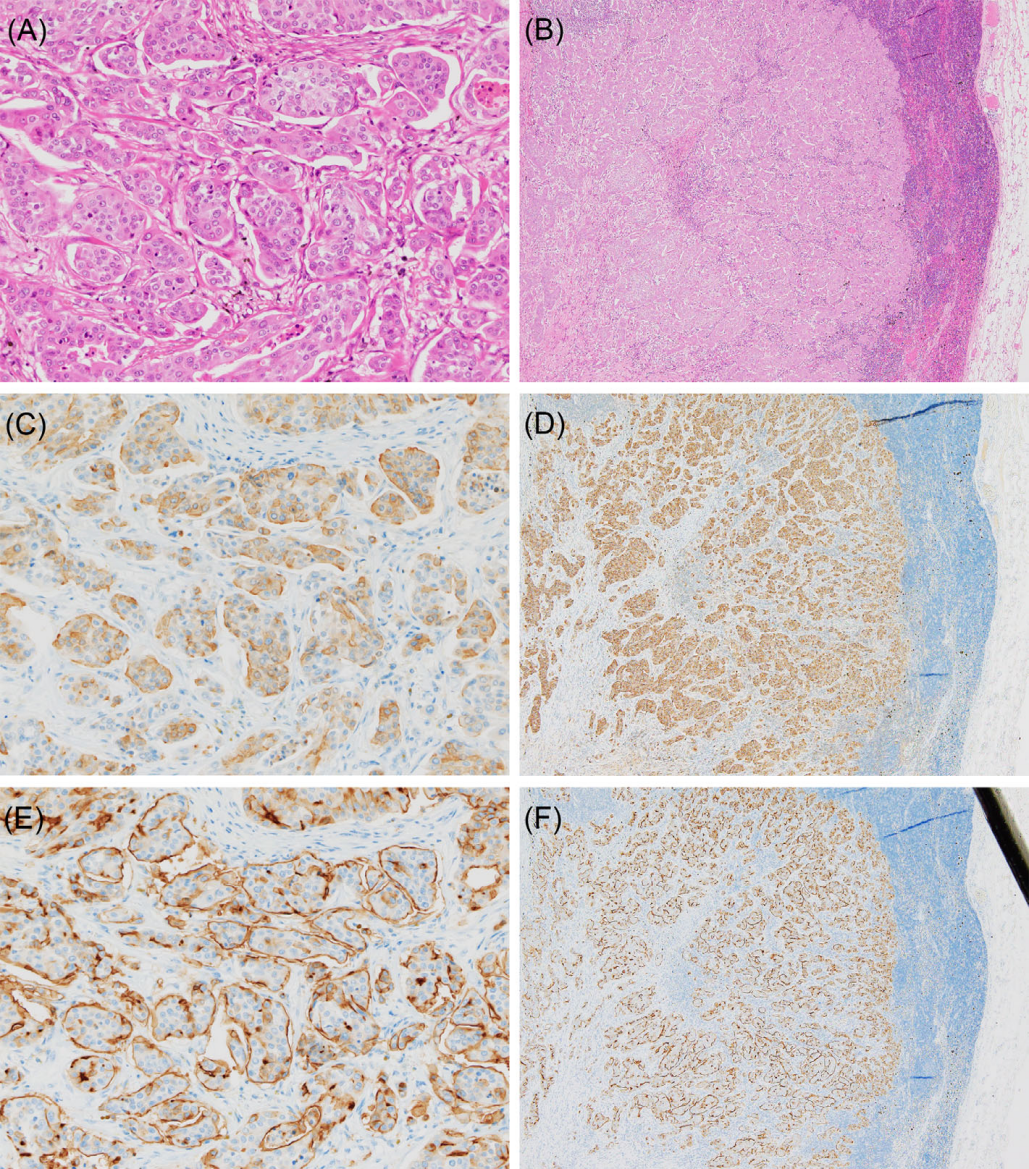
Pathological findings of neuroendocrine mammary neoplasm with invasive micropapillary pattern and nodal metastasis. Serial histological sections from primary (A, C and E: x200) and metastatic (B, D and F: x40) sites demonstrate distinct dual neuroendocrine and micropapillary morphologies [H&E (A and B)] with immunohistochemically verified neuroendocrine nature [chromogranin A (C and D)] and “inside‐out” features [MUC1 (E and F)] of this tumor. (microscope: Olympus BX53 with UPlanSApo, camera: Olympus DP74)

A well‐demarcated, multinodular, red‐brown tumor, which measured 15 × 15 × 15 cm, was found in the specimen obtained at breast removal. Histologically, this solid and cystic lesion consisted of medullary growth of carcinoma cells accompanied by a highly developed vascular or fibrovascular stroma and remarkable hemorrhage. The carcinoma cell nests varied in size, displaying a retraction artifact as well as antipolarity (Figure 1). The carcinoma cells were polygonal, occasionally showing a short spindle shape, and possessed finely granular, relatively eosinophilic cytoplasm and round‐to‐ovoid, fine‐granular nuclei (Figure 1A). Eighteen mitotic figures were counted in 10 high‐power fields. The histological grade was estimated to be 2. No conventional in situ carcinoma component was observed. Macrometastases, up to 13 × 8 mm in size, showing the same morphological features as the primary site (Figure 1B), were present in 3 of 15 dissected ipsilateral axillary lymph nodes.

The TNM stage diagnosis of pT3, pN1a, cM0, Stage IIIA, was definitely made.

Immunohistochemical examinations revealed primary and metastatic carcinoma cells to be diffusely positive for chromogranin A (Figure 1C, D) and the estrogen receptor (ER) (Allred's total score: 8) and to be focally reactive for synaptophysin and the progesterone receptor (PgR) (Allred's total score: 5). HER2 and cytokeratin 5/6 were negative, and the Ki‐67 (MIB‐1) labelling index was 36.2% at the hot spot. Band‐like expressions of MUC1 (Figure 1E, F) and EMA were noted on the stroma‐facing surface of the carcinoma cell clusters, accentuating the outlines of the micropapillary units. The primary antibodies used are detailed in Table 1.

**TABLE 1:** Panel of immunohistochemical antibodies used in this report

Antibody		Clone	Source	Dilution
Chromogranin A	Mouse monoclonal	LK2H10	Roche Diagnostics, Mannheim, Germany	Ready‐to‐use
Synaptophysin	Mouse monoclonal	27G12	Nichirei Bioscience Inc., Tokyo, Japan	Ready‐to‐use
Estrogen receptor	Rabbit monoclonal	SP1	Roche Diagnostics, Mannheim, Germany	Kit
Progesterone receptor	Rabbit monoclonal	1E2	Roche Diagnostics, Mannheim, Germany	Kit
HER2	Rabbit monoclonal	4B5	Roche Diagnostics, Mannheim, Germany	Kit
Ki‐67	Mouse monoclonal	MIB‐1	Dako, Copenhagen, Denmark	1:50
Cytokeratin 5/6	Mouse monoclonal	D5/16 B4	Dako, Copenhagen, Denmark	1:100
MUC1	Mouse monoclonal	DF3	Fujirebio Diagnostics, Inc., Malvern, PA, United States	1:10
EMA	Mouse monoclonal	E29	Dako, Copenhagen, Denmark	Ready‐to‐Use
Somatostatin receptor 2	Rabbit monoclonal	UMB1	Abcam, Cambridge, UK	1:200

Anastrozole (1 mg daily), an oral aromatase inhibitor, is currently being administered as adjuvant endocrine therapy, while chemotherapy was not concomitantly given, despite the relatively high MIB‐1 index, based on patient background factors such as recovery from the brain infarct. She remains alive and well, 12 months after surgery, with no evidence of recurrence or metastasis.

## 3 DISCUSSION

Intriguingly, the tumor in our present case had, pathologically, simultaneous neuroendocrine and micropapillary features, that is, relatively small nests composed of polygonal to shortly fusiform cancer cells with finely granular, eosinophilic cytoplasm and ovoid, fine‐granular nuclei displaying an “inside‐out” structure surrounded by empty spaces. [Correction added on ** January 2023, after first online publication: The preceding sentence has been removed and now added in this version.] Furthermore, immunohistochemistry for specific markers such as chromogranin A and MUC1 reasonably verified the nature and morphological features of the tumor. This is the first report, according to our extensive literature search, to describe a case with a mixed neuroendocrine‐micropapillary neoplasm of the breast.

The prognosis of patients with mammary NENs is still controversial: some investigators demonstrated this tumor entity to generally show rather indolent biological behavior, with higher response rates to both hormonal treatments and chemotherapy,^3^ whereas others recently reported that NEN is a distinctive type of aggressive IBC,^4^ though most show ER and/or PgR expression without HER2 gene amplification.^5^, ^6^, ^7^, ^8^, ^9^ Furthermore, multivariate analyses have revealed that, in patients with NENs of the breast, overall survival can be predicted by tumor size, lymph node status, and the MIB‐1 proliferation rate.^10^ On the other hand, “micropapillary structure” of neoplastic cells in the stromal tissue, regardless of the proportion of its component or the original site, is associated with an affinity for lymphatic vessels, resulting in a high prevalence of nodal involvement.^11^, ^12^, ^13^, ^14^ Our present patient with an unusual breast cancer showing both invasive micropapillary and neuroendocrine features developed plural lymphoglandular metastases as well as having a tumor with a large diameter and luminal B‐like immuno‐profile. Accordingly, meticulous clinical follow‐up continues to be essential for this case.

From the perspective of new treatments, in the recent years, somatostatin receptor type 2A has also attracted academic attention as it is both an important targeted therapy and a diagnostic procedure in the breast neuroendocrine oncology field.^7^ However, unfortunately, distinct receptor immuno‐expression was not obtained in our current case (data not shown). Focusing on invasive micropapillary carcinoma, effective surgical intervention frequently requires extended margins as well as vigilant preoperative axillary staging due to an increased incidence of lymph node involvement and locoregional recurrence.^13^, ^14^ The latest foundation study revealed a multitude of adherence molecules and chemotactic factors, including interleukin 1‐β, N‐cadherin, SDF‐1/CXCR4, and several others, involved in the histological tendency for lymphatic permeation.^13^ Future investigations based on translational research might include the application of novel molecules as therapeutic targets or prognostic markers.

Preliminary results from this article were published as a poster presentation at the XXXIV International Congress of the International Academy of Pathology (Sydney, Australia, October 2022)^15^ and the 34th European Congress of Pathology (Basel, Switzerland, September 2022).^16^

## AUTHOR CONTRIBUTIONS

**Tomonori Kawasaki:** Conceptualization (equal); data curation (equal); formal analysis (equal); funding acquisition (lead); investigation (equal); methodology (equal); project administration (equal); resources (lead); supervision (equal); validation (equal); visualization (equal); writing – original draft (lead); writing – review and editing (equal). **Tomoaki Tashima:** Conceptualization (equal); data curation (equal); formal analysis (equal); investigation (equal); validation (equal); visualization (equal); writing – review and editing (equal). **Chisako Muramatsu:** Data curation (equal); formal analysis (equal); investigation (equal); methodology (equal); validation (equal); visualization (equal). **Akihiro Fujimoto:** Data curation (equal); formal analysis (equal); investigation (equal). **Yoko Usami:** Formal analysis (equal); validation (equal); visualization (equal). **Hitomi Kodama:** Investigation (equal); methodology (equal); validation (equal). **Jiro Ichikawa:** Data curation (equal); visualization (equal); writing – review and editing (equal). **Hirokazu Nagai:** Project administration (equal); supervision (equal); writing – review and editing (equal). **Kiyomi Taniyama:** Conceptualization (equal); project administration (equal); supervision (equal); writing – review and editing (equal).

## FUNDING INFORMATION

Grants‐in‐Aid for Scientific Research from the Japanese Ministry of Education, Culture, Sports, Science and Technology, Grant/Award Numbers: 20K08131, 21K06910; National Hospital Organization (NHO) Grant, Grant/Award Number: H29‐NHO‐01; and Saitama Medical University Internal Research Grants.

## CONFLICT OF INTEREST

The authors state that there are no conflicts of interest to disclose.

## ETHICS STATEMENT

The study was conducted in accordance with the Declaration of Helsinki and approved by the Institutional Review Board of Saitama Medical University International Medical Center (Approval No. 2021–077). Informed consent has been obtained from the patient, and the paper does not contain any identifying information pertaining to the patient.

## Data Availability

The data that support the findings of this study are available on request from the corresponding author. The data are not publicly available due to privacy or ethical restrictions.
